# Study partners should be required in preclinical Alzheimer’s disease trials

**DOI:** 10.1186/s13195-017-0327-x

**Published:** 2017-12-06

**Authors:** Joshua D. Grill, Jason Karlawish

**Affiliations:** 1 Departments of Psychiatry and Human Behavior and Neurobiology and Behavior, Institute for Memory Impairments and Neurological Disorders, 3204 Biological Sciences III, University of California, Irvine, CA 92697 USA; 2 Departments of Medicine, Medical Ethics and Health Policy, and Neurology, Penn Memory Center, University of Pennsylvania, Philadelphia, PA USA

**Keywords:** Preclinical Alzheimer’s disease, Informant, Clinical trial, Biomarker, Study partner

## Abstract

**Background:**

In an effort to intervene earlier in Alzheimer’s disease (AD), clinical trials are testing promising candidate therapies in preclinical disease. Preclinical AD trial participants are cognitively normal, functionally independent, and autonomous decision-makers. Yet, like AD dementia trials, preclinical trials require dual enrollment of a participant and a knowledgeable informant, or study partner.

**Main text:**

The requirement of dyadic enrollment is a barrier to recruitment and may present unique ethical challenges. Despite these limitations, the requirement should continue. Study partners may be essential to ensure participant safety and wellbeing, including overcoming distress related to biomarker disclosure and minimizing risk for catastrophic reactions and suicide. The requirement may maximize participant retention and ensure data integrity, including that study partners are the source of data that will ultimately instruct whether a new treatment has a clinical benefit and meaningful impact on the population health burden associated with AD. Finally, study partners are needed to ensure the scientific and clinical value of trials.

**Conclusions:**

Preclinical AD will represent a new model of care, in which persons with no symptoms are informed of probable cognitive decline and eventual dementia. The rationale for early diagnosis in symptomatic AD is equally applicable in preclinical AD—to minimize risk, maximize quality of life, and ensure optimal planning and communication. Family members and other sources of support will likely be essential to the goals of this new model of care for preclinical AD patients and trials must instruct this clinical practice.

## Background

In the US and other developed nations, the disability caused by Alzheimer’s disease (AD) dementia exacts tremendous costs on patients and their families, and population aging is increasing these costs [1]. One strategy to address these costs is to discover therapies that slow the onset and progression of disability. This strategy, together with advances in understanding AD biology, has led to trials that enroll volunteers with no cognitive or functional impairments but who have genetic or biomarker evidence that suggests they are at risk of developing AD dementia. These trials are an essential part of validating a new stage of AD called “preclinical AD,” a term that describes the presence of pathophysiology without cognitive or functional impairments [2, 3].

Preclinical AD treatment development faces several challenges. The most immediate are barriers to the efficient conduct of randomized controlled trials. Several design features deter enrollment. These include a long time commitment (typically 3 to 5 years), burdensome procedures (frequent brain imaging), compounds with notable risks (such as brain edema), and the requirement to learn AD genetic or biomarker test results that carry uncertain prognosis and substantial ethical, legal, and social consequences [4–7]. Finally, all preclinical AD trials require dual enrollment. In addition to the participant, a knowledgeable informant must consent to attend study visits and complete outcome measures. This person is commonly called the “study partner.” Without a study partner, an otherwise eligible subject cannot be enrolled.

In AD dementia clinical trials, caregivers typically serve in the study partner role and are vital to trial success. They drive dementia trial enrollment decisions [8] and ensure informed consent [9], protocol compliance, and study completion [10]. They provide data used to determine treatment efficacy [11]. In contrast, subjects in preclinical AD trials are functionally independent and so able to choose to enroll, provide their own informed consent, and comply with study procedures. They may be unable or unwilling to identify a study partner [12]. Moreover, the primary outcomes in current preclinical AD trials are informant-independent measures of cognition [13, 14]. These points add up to a compelling case. The study partner requirement is a barrier to efficient enrollment and timely study completion that delays the goal of discovering an effective prevention. Why then, should preclinical AD trials require study partners?

The answer to this question has substantial implications not only for the success of trials, but also for the successful translation and dissemination of the preclinical AD diagnosis from research into clinical practice. Although the study partner role in preclinical AD trials is different than in dementia trials and may slow the progress of drug discovery, the role is essential.

In this article, we argue that preclinical AD trials should enroll study subjects and study partners. This “pre-clinical dyad” is both ethically and scientifically necessary to ensure trial success and to instruct future clinical practice. We close by outlining the research essential to instruct investigators and clinicians on how best to work with this novel and important dyad.

### The study partner requirement is essential to ensure participant safety and well-being

To minimize cost, maximize efficiency, and understand the clinical impact of knowledge of AD gene or biomarker results, most preclinical AD trials use a “transparent” enrollment design [15]. In transparent designs, the investigator discloses AD genetic or biomarker test results to the subject [16]. Standardized approaches to do this and to monitor participant health and safety after disclosure have been developed [17, 18]. Prospective safety data remain limited, however, as many of the first transparent design studies are ongoing. To date (September 2017), no study has reported a safety concern related to the impact of learning gene or biomarker results.

Data are available from two studies that have investigated the safety of disclosing amyloid positron emission tomography (PET) AD biomarker information to cognitively normal participants [19, 20]. In their randomized preclinical AD trial of physical exercise, Burns and colleagues required disclosure as part of enrollment after excluding participants demonstrating either depression or anxiety [20]. The investigative team disclosed results at a unique study visit, describing the outcome of PET imaging as demonstrating elevated or not elevated amyloid levels. Lim and colleagues made qualitative PET results (described as positive or negative) available to participants’ neurologists as an ancillary study to a preclinical AD trial enrolling older participants with both a family history of AD and subjective memory complaints [19]. Participants had the option to learn their results through their physician. Both studies were relatively small (27 and 4 amyloid-positive individuals in the Burns and Lim studies, respectively) and found that disclosure was largely safe; low rates of psychological adverse events were observed through standardized outcome measures. In both studies, however, participants learning positive amyloid PET results experienced elevations in distress and anxiety at the time of disclosure, which were deemed not clinically significant. In the Burns study [20], participants were required to enroll with a study partner. In the Lim study [19], all participants indicated that they had shared their results with family or friends and reported satisfaction with their support network.

Similar findings of test-related distress exist for the disclosure of AD genetic results. The Risk Evaluation and Education in AD (REVEAL) studies have examined the safety of disclosing apolipoprotein E (APOE) ε4 allele genotypes to cognitively normal middle-aged adults with a first-degree family history of AD. Learning AD genetic risk did not cause clinical anxiety or depression, but individuals who learned that they were ε4 carriers experienced transient test-related distress [21]. Although participants were not required to enroll with a study partner, discussing test results with others was associated with reduced scores on depression and anxiety measures 1 year after APOE disclosure [22]. APOE test results were most commonly shared with spouses or other family members [23].

These data suggest that study partners may play a key role in how participants in preclinical AD trials cope with anxiety and distress, and that enrolling participants who lack a support network could exacerbate the psychological harms of AD risk disclosure. Surveys of the public and of registries of individuals willing to participate in AD research find that 10–12% of individuals wish to gain access to biomarker and/or genetic risk information to instruct suicide planning [24–26]. One of these studies found that suicide planning was associated with feelings of non-support and being single [24]. Investigators are ethically bound to minimize risk, maximize benefit, and ensure the safety of those enrolling in trials; enrolling only participants with a satisfactory support network, including at least one individual who can serve as study partner, is a sensible means to achieving this ethical obligation.

### The study partner requirement is essential to trial validity

Preclinical AD trials use objective cognitive tests as primary outcomes. Key secondary outcomes, however, assess subjective cognitive and functional performance. It is unclear whether subjects or their informants provide the more accurate assessment of these constructs. Traditional measures of global or functional performance, such as the Clinical Dementia Rating (CDR) scale [27], are being used in preclinical AD trials. These rely on study partner reports. Other scales, such as those developed in the AD Cooperative Study Prevention Instrument (ADCS PI) study [28], include participant and study partner versions, enabling direct comparisons to determine which is optimal for preclinical AD trials.

The ADCS Activities of Daily Living Prevention Instrument (ADCS-ADL-PI) scale [29] examines 15 instrumental ADLs and 5 physical functions. In the ADCS PI study, the participant version demonstrated greater sensitivity to age effects, but also showed an apparent racial bias [29]. Both versions showed modest sensitivity for identifying global and cognitive decline, though only 12-month follow-up data have been reported (the duration of the study was 48 months) [29]. Both versions are used in the Anti-Amyloid treatment in Asymptomatic AD (A4) study [30], and study partners are being asked additional questions about the frequency of functional tasks and the time to complete those tasks. The decision to rely on study partners for these additional items is supported by observations that in mild cognitive impairment (MCI), study partner reporting of functional impairment is more strongly predictive of conversion to dementia than is self-reporting [31].

The Cognitive Function Instrument (CFI) was also developed in the ADCS PI study [32]. The CFI incorporates 14 subjective items assessing cognitive performance and has been proposed as a potential functional outcome measure for preclinical AD trials [33]. In the ADCS PI study, participant self-ratings were more closely related to objective cognitive testing performance than were study partner ratings at baseline. Study partner ratings were better correlated at 48 months [33]. The combination of participant and partner reports was more strongly correlated with cognitive test performance than either report alone [33]. Similar results were observed in the National Alzheimer’s Coordinating Center Uniform Data Set; subjective complaints in both participants and their study partners were associated with greater risk for MCI than were subjective complaints by either the participant or the study partner alone [34]. These results suggest that study partner reporting may be essential to maximize data integrity in preclinical AD trials.

Study partners have key roles in assuring the validity of other aspects of preclinical AD trials. One role is minimizing missing data by preventing drop out. Preclinical AD trials are lengthy and participation can be burdensome, requiring many complex visits. Previous AD prevention trials have incurred greater than expected dropout [35], putting statistical power at risk. In both AD dementia [36] and MCI trials [37], participants lacking a spouse are at increased risk for dropout. As with trials in individuals with cognitive impairment, preclinical AD trial participants who lack a support network such as a study partner may be at increased risk for dropout.

### The study partner requirement is essential to ensure the scientific and clinical value of preclinical AD trials

Based on US Food and Drug Administration (FDA) guidance, AD dementia trials assess efficacy using dual primary outcomes—typically a measure of cognitive performance and a measure of global or functional performance. The latter is required to demonstrate the clinical benefit of the cognitive performance. The first AD dementia trials used instruments based only on clinician assessment of the patient to assess clinical meaningfulness [38]. Expert consensus [38] and research demonstrating the benefit of informant reporting [39], however, led to a state of the art in which AD dementia trial co-primary outcomes incorporate or exclusively rely upon study partner reporting.

In preclinical AD, FDA guidance indicates that reducing decline on a single primary outcome measure may be sufficient to achieve approval [40], with post-approval studies to confirm that treatments result in clinically meaningful functional benefit. Though novel approaches to demonstrating clinical benefit should be pursued, such as assessing resource utilization through medical record or claims data [41], long-term extension studies using traditional global or functional outcome measures seem the most probable approach. These outcome measures require a study partner. Study partner-based tools will also be vital to examining the public health implications of preclinical AD treatment, including the time to dementia onset, the number of dementia cases, and the economic burden of disease [42].

Preclinical AD trials will change how society and medicine conceive of what is AD [4]. These trials must instruct not only the use of new therapies, such as drug dosage and safety, but also how to provide care in this new model of AD. In the absence of a drug that halts the onset of cognitive decline across all patients, some patients will suffer cognitive impairment. AD will remain a disease that requires planning, support, and care. No standards exist for this clinical practice in persons who have preclinical AD. Trials should be an essential source of data to develop and refine this practice.

Trial results should instruct the clinical practice of widescale biomarker and genetic testing and disclosure. Truth and honesty in diagnostic disclosure, no different from that recommended in AD dementia [43], includes communicating the prognosis of cognitive decline and functional impairment. Discussing this information will necessarily engender other conversations to make plans to further reduce the risk of cognitive decline, such as exercise and cardiovascular health. Patients will likely begin to consider financial matters such as the timing of retirement and where they will live. Issues of future healthcare decisions will be considered [44]. Such planning will be critical not only to maximize health, but also to minimize the risks associated with early signs of cognitive impairment such as medication errors, driving accidents [45], and financial error and abuse [46].

A sensible means to do all of this is to involve another person in the life of a patient with preclinical AD. This person should be the study partner. Patients should want to have someone, such as a partner, adult child, or close friend, to help them to plan for this future. Patients may need to have someone to watch over them because, in time, as preclinical becomes clinical AD, patients will need the help of someone else to manage their problems. Physicians providing clinical care for persons with preclinical AD will strongly recommend the involvement of such a person in both diagnostic and treatment phases of management. Filling this role, or helping to identify a network that can support these needs, should be part of the role of a study partner.

## Areas in need of research

### Ensuring that the benefits of preclinical research apply to all older people at risk for AD dementia

Many potentially eligible preclinical AD trial participants lack a person who can fill the study partner role. While this may effectively limit the pool of participants, the requirement may also produce sample bias. Understanding the implications of this bias and ensuring that those who lack the support network needed to enroll in trials can still benefit from the knowledge gained through them will be necessary to maximize the public health impact of preclinical AD research.

### Current study partner inclusion criteria do not guarantee knowledgeable informants

The relationship between criteria to serve as study partner and the integrity of informant data has not been established in preclinical AD, or in AD dementia for that matter. In AD dementia, spousal study partners differ from non-spousal study partners in the accuracy of cognitive assessments [47] and in their concordance with patient ratings of quality of life [48]. No study of informant assessment of cognitive function in preclinical AD has explored the qualifications of the study partner and whether some individuals may yield more sensitive identification of participant cognitive decline than others. Requiring study partners will preclude some potentially eligible participants from enrolling. Ensuring that those who are enrolled will provide high-integrity data will be essential to safeguarding trial value and justifying the cost of the requirement.

### The study partner requirement may cause unique ethical risks

A critical aspect of preclinical AD trial conduct is to protect participants from unwanted disclosure of genetic or biomarker information through electronic medical records [4]. However, the risk for stigma in the home and in social situations remains in these trials and one open question is how often and with whom do preclinical AD trial participants share their testing results?

In a study of preclinical AD trial enrollment decisions, in which participants were randomly assigned to consider a hypothetical trial that did or did not require biomarker disclosure, we found that the study partner requirement was a more important barrier to enrollment when disclosure was required [12]. The requirement was rated as more important than drug risks in the disclosure arm of this study. These preliminary data suggest that preclinical AD trial participants may be reluctant to share with others that they have biomarker evidence of AD. Some participants may face an unenviable choice: have others potentially learn information about their health they do not want shared, or forego enrolling in a study in which they wish to participate.

Further study will be necessary to instruct optimal means to overcome the study partner requirement as a barrier to enrollment without sacrificing participant privacy and confidentiality. Greater understanding of the frequency and extent of this occurrence are needed. Modifying or improving the disclosure process, which does not currently emphasize the role of the study partner, may improve the willingness of participants to share biomarker information. Ensuring that participants are comfortable and ready to share AD risk information may be critical, as may be education and counseling of others in their support network.

Respecting participant autonomy is an ethical requirement in clinical research. Requiring preclinical AD trial participants to involve another person in the study does not disrespect autonomy. If this requirement does not align with a person’s values of identity, privacy, and authority, he or she can freely choose not to enroll. Among those who do enroll, the requirement may foster their autonomy. These participants are at risk for cognitive and functional impairments. A study partner may become the participant’s trusted advocate to whom the participant can tell how they would like to be cared for and other plans for the future. The advocate can also monitor the participant for signs of impairment. These activities are recognized as a means to maintain autonomy despite the loss of capacity.

### Helping study partners become caregivers

A vast scientific literature on the caregiver has informed interventions to improve the lives of patients with AD dementia [49]. Some preclinical AD participants will become clinical AD patients and need a caregiver. Little is known about the implications of who is available to preclinical AD patients for support, to aid with planning, and for assistance with instrumental ADLs with high cognitive demand. The field will ultimately need to know who these people are, if and when they change, and what if any characteristics predict their performance in these roles. Moreover, these individuals will likely need interventions to assist them in these roles, not unlike the interventions to assist dementia caregivers.

## Conclusions

The imperative to reduce the public health burden of AD and the field’s growing consensus that early intervention may be essential to slowing disease progression has launched a novel field of investigation, preclinical AD trials. The optimal design features for these trials remain areas of active study and debate, ranging from whether biomarker information can or should be disclosed to participants, to the number and types of outcome measures that should be used to assess drug efficacy. We examined one particular aspect of preclinical AD trial designs that has scientific, regulatory, and ethical implications—the requirement for a study partner (Table 1).

**Table 1:** Arguments for and against requiring study partners in preclinical Alzheimer’s disease trials

Against dyadic enrollment	In favor of dyadic enrollment
• The requirement is a barrier to enrollment	• Study partners may be necessary to ensure participant safety, assisting in overcoming distress related to biomarker disclosure
• Study partners are not needed to ensure adequate informed consent since participants are cognitively normal, autonomous decision makers	• Individuals who lack a study partner may be at greatest risk for catastrophic reaction, including suicide
• The requirement may introduce novel risks related to confidentiality/privacy	• The study partner can provide support that will mitigate stigma
• The requirement may introduce the risk of stigma to participants	• The requirement may maximize participant retention
	• The requirement may optimize data integrity
	• Scales to measure patient function require study partners
	• Involving study partners in trials may best instruct an as yet undefined practice

AD is an insight-robbing neurodegenerative disease. Trial participants themselves, therefore, may not be the best judge of clinically important changes in their cognition and function. However, disclosure of AD biomarkers or genes carries risks to participants, such as stigma and discrimination. Together, these points create an ethically complex and unique situation. Whereas other chronic and progressive diseases have similar rationale for earliest possible identification of disease onset, such as genetic and biomarker tests for cancers, these diseases do not generally affect cognition nor are they associated with stigmas such as “loss of self” or “loss of personhood.” Rather, preclinical AD trials are likely to provide important guidance for trials in other neurological conditions in which presymptomatic diagnosis may optimize treatment efficacy [50].

The requirement of dual enrollment of participants and knowledgeable study partners will be essential to ensuring the integrity of preclinical AD trial data, minimizing loss-to-follow-up, and ensuring participant safety. Safety is paramount in trials in this nascent diagnostic construct. Moreover, study partner reporting will be essential for assessing clinical effectiveness and public health outcomes of preclinical treatment.

Further study is needed to understand how the study partner requirement may create challenges to efficient enrollment. The costs of recruiting subjects and their study partners, however, are secondary to the value of knowledge that will discover a novel clinical practice. Trials have a special role to develop and refine this knowledge. Preclinical AD care will surely be more than ordering a biomarker test, prescribing a drug, and scheduling follow-up in some time interval. Patients in the early stages of AD require counseling, planning assistance, and other supportive services. Preclinical AD trials must be designed to instruct this future practice.

## Abbreviations

A4: Anti-Amyloid treatment in Asymptomatic AD
AD: Alzheimer’s disease
ADCS: AD Cooperative Study
ADL: Activities of daily living
APOE: Apolipoprotein E
CDR: Clinical Dementia Rating scale
CFI: Cognitive Function Instrument
FDA: Food and Drug Administration
MCI: Mild cognitive impairment
PET: Positron emission tomography
PI: Prevention instrument
REVEAL: Risk Evaluation and Education in AD
